# *In Vitro* Modelling of *Chlamydia trachomatis* Infection in the Etiopathogenesis of Male Infertility and Reactive Arthritis

**DOI:** 10.3389/fcimb.2022.840802

**Published:** 2022-01-31

**Authors:** Simone Filardo, Marisa Di Pietro, Fabiana Diaco, Rosa Sessa

**Affiliations:** Department of Public Health and Infectious Diseases, Section of Microbiology, University of Rome “Sapienza”, Rome, Italy

**Keywords:** *Chlamydia trachomatis*, obligate intracellular bacteria, human prostate cells, human Sertoli cells, human synovial cells

## Abstract

*Chlamydia trachomatis* is an obligate, intracellular bacterium responsible for a range of diseases of public health importance, since *C. trachomatis* infection is often asymptomatic and, hence, untreated, leading to chronic complications, including prostatitis, infertility, and reactive arthritis. The ample spectrum of diseases caused by *C. trachomatis* infection is reflected in its ability to infect and multiply within a wide range of different cell types. Cervical epithelial cells, to date, have been the most studied cellular infection model, highlighting the peculiar features of the host-cell inflammatory and immune responses to the infection. Herein, we provide the up-to-date evidence on the interaction between *C. trachomatis* and human prostate epithelial, Sertoli and synovial cells.

## Introduction

*Chlamydia trachomatis*, obligate, intracellular bacterium responsible for a range of diseases of public health importance, is the leading cause of sexually transmitted bacterial infection worldwide, with estimates of more than 130 million new cases each year (Rowley et al., 2019). In women, the most common clinical manifestations are cervicitis and urethritis, whereas in men these are urethritis and epididymitis, although in the majority of cases *C. trachomatis* genital infection is asymptomatic and, hence, untreated, leading to chronic complications, including prostatitis, infertility, and reactive arthritis (ReA) (O’Connell and Ferone, 2016; Di Pietro et al., 2019).

Prostatitis, one of the most common urologic problems for men younger than 50 years, and risk factor for infertility (Khan et al., 2017), has a prevalence of approximately 8% to 16%, and around 5% to 10% of all cases have a bacterial origin, with *C. trachomatis* involved in up to 27% of all bacterial infections of the prostate (Ostaszewska et al., 1998; Badalyan et al., 2003; Krieger et al., 2008; Ouzounova-Raykova et al., 2010; Trinchieri et al., 2021). Nevertheless, chronic bacterial prostatitis is frequently underestimated because urinary tract infections often remain undocumented and thus neglected (Ouzounova-Raykova et al., 2010). Male infertility also remains a neglected area in sexual and reproductive health although it has a significant impact on public health worldwide, since approximately 15-20% of reproductive age couples are infertile in industrialized countries, and, in 30% of all cases, fertility problems are solely due to the male partner, with around 15% of idiopathic cases attributed to infectious causes (Henkel et al., 2021; Thoma et al., 2021). Similarly, ReA is a frequently misdiagnosed condition, due to difficulties in relating past infections with compromised joint functions. It is estimated that approximately 4–8% of patients will develop ReA one to six weeks after a urogenital *C. trachomatis* infection, and in 30% of all cases, ReA persists for years, leading, eventually, to joint deformities and ankylosis (Zeidler and Hudson, 2016; Di Pietro et al., 2019; Henkel et al., 2021).

The ample spectrum of diseases caused by *C. trachomatis* infection is reflected in its ability to infect and multiply within a wide range of different cell types, such as cervical epithelial cells, peripheral blood mononuclear cells (Dolat and Valdivia, 2019; Lausen et al., 2019). The typical model of chlamydial intracellular development has been mostly investigated in human cervical epithelial cells (HeLa) and murine fibroblasts (McCoy), for which *C. trachomatis* possesses the highest tropism (Belland et al., 2003; Guseva et al., 2007; Vromman et al., 2014; Petyaev et al., 2017; Sessa et al., 2017a; Filardo et al., 2019a; Liang and Mahony, 2019; Jøraholmen et al., 2020). *C. trachomatis* developmental cycle occurs entirely within a cell-derived membrane bound vesicle termed inclusion, where Chlamydiae alternate between the elementary body (EB), the extracellular and infectious form, and the reticulate body (RB), the metabolically active form, responsible for intracellular replication (AbdelRahman and Belland, 2005). The first stage of chlamydial developmental cycle consists in EBs adhesion to host cell membrane receptors, like glycosaminoglycans (Conant and Stephens, 2007). Then, chlamydial EBs enter the host cell by endocytosis, *via* a two-step process involving a reversible interaction mediated by heparin-sulphate proteoglycans followed by irreversible binding to host receptors (Conant and Stephens, 2007). Soon after attachment to the host cell, EBs are internalized and confined to the inclusion, where they differentiate to RBs; within 24 hours post-infection (h.p.i.), chlamydial RBs replicate by binary fission (Bastidas et al., 2013). As inclusion expands, approximately 24-48 h.p.i., the majority of RBs begin to transition back to EBs in an asynchronous process (AbdelRahman and Belland, 2005). At the end of the developmental cycle, at about 48 h.p.i., the EBs are finally released from the host by cell lysis or extrusion (Yang et al., 2015; Zuck et al., 2016). Thereafter, a multitude of infectious EBs spread and infect neighboring cells, perpetuating the infectious process.

To date, the human cervical epithelial cell has been the most studied cellular infection model, focusing on chlamydial growth and on the peculiar features of the host-cell inflammatory and immune responses to the infection (Lad et al., 2005; Sessa et al., 2017b; Tang et al., 2021). Following chlamydial infection, the host cell response typically begins with the activation of a complex network of immune receptors (TLR2 and TR4) and their respective downstream signaling pathways (myeloid differentiation primary response 88, MyD88, and nuclear factor kappa-light-chain-enhancer of activated B cells, NFkB) (O’Connell et al., 2006; Sellami et al., 2014). This results in the induction of proinflammatory cytokines, involved in either the elimination of *C. trachomatis* or tissue damage related to chronic inflammatory state, including interleukin (IL)-1α, IL-6, IL-8 and interferon (IFN)-γ (O’Connell et al., 2006; Rey-Ladino et al., 2014; Sellami et al., 2014). IFN-γ, in particular, has been identified as a major player in the clearance and protection against *C. trachomatis* infection, by modulating a plethora of host cell signalling pathways, like the activation of NF-kB and the inflammasome network (Rothfuchs et al., 2004; Webster et al., 2017).

Overall, the pathogenic mechanisms underlying *C. trachomatis*-mediated chronic complications have received the most research attention in women, whereas chlamydial survival strategies as well as the host defense pathways, involved in the onset and development of prostatitis, male infertility, and reactive arthritis, are now beginning to emerge. Therefore, herein we provide the up-to-date evidence on the interaction between *C. trachomatis* and human prostate epithelial, Sertoli and synovial cells.

## *C. trachomatis* Infection Models in Prostatitis, Male Infertility and Reactive Arthritis

### Human Prostate Epithelial Cells

The first evidence to demonstrate *C. trachomatis* growth within primary human prostate epithelial cells came from Greenberg et al, in 1985 (Greenberg et al., 1985). Since then, few studies on chlamydial interaction with prostate epithelial cells were performed, describing both the ability of *C. trachomatis* to replicate in these cells and the specific host-cell inflammatory and immune pathways in response to the infection. Specifically, a study investigating the inflammatory profile of *C. trachomatis* infection in humane prostate epithelial cells (PNT2) and urethral epithelial cells (THUEC), demonstrated that prostate epithelial cells produced larger quantities of IL-6 and IL-8 than urethral epithelial cells, suggesting the increased levels of these cytokines as possible markers for *C. trachomatis* infection of the prostate (Al-Mously and Eley, 2007).

At a later time, a potential link between the inflammatory damage and *C. trachomatis* infection of the prostate was suggested, since a strong pro-inflammatory response, characterized by NFkB activation and TLR2/TLR4 upregulation, was observed, leading to increased inflammatory cytokine expression, like IL-6, IL-8, IL-1β and tumor necrosis factor (TNF)α (Sellami et al., 2014). Lastly, a recent study has described the efficient propagation of *C. trachomatis* in a malignant prostate epithelial cell line (CWR-R1) (proportion of infected cells 28.1%) accompanied by enhanced transcription of IL-6 and fibroblast growth factor (FGF)-2 genes, encoding two important pro-inflammatory cytokines involved in the progression of prostate cancer (Petyaev et al., 2019).

### Human Sertoli Cells

In recent years, a study employing a murine model has postulated the direct infection of the seminiferous tubule epithelium, formed by Sertoli cells, as an interesting pathophysiological mechanism for *C. trachomatis*-mediated male infertility, leading to compromised spermatogenesis with reduced sperm count, motility and altered morphology of mature spermatozoa (Bryan et al., 2021).

On this basis, we have investigated, for the first time, the interaction between *C. trachomatis* and human primary Sertoli cells *in vitro*, demonstrating a distinct growth profile of *C. trachomatis*, with a very long eclipse period (after 36 h.p.i.), the appearance of infectious EBs beyond 48 h.p.i., the persistence of inclusions up to 96 h.p.i. and a low infection efficiency (Filardo et al., 2019b). This greatly differed from the chlamydial growth cycle as typically seen in cervical epithelial cells, where the transition of RBs to EBs happen after 22 h.p.i. and the release of infectious EBs from host cells is usually observed 36 to 48 h.p.i. (Guseva et al., 2007; Vromman et al., 2014; Skilton et al., 2018). Of great pathological importance, the development of *C. trachomatis* inclusions has also been demonstrated to visibly damage the host cell cytoskeleton, as shown by the reorganization of Vimentin-based intermediate filaments and α-tubulin microtubules in thick fibres surrounding chlamydial inclusions (Filardo et al., 2019b). This, in turn, might alter the integrity of the blood-testis barrier, a structural compartment of the seminiferous tubules essential for germ cell development and maturation, impairing the spermatogenesis and contributing to male infertility (Mruk and Cheng, 2015). On this regard, the evidence that human spermatozoa were unaffected by biomolecules produced by chlamydial infected Sertoli cells, suggests that *C. trachomatis* is more likely to influence the early stages leading to the development of mature spermatozoa.

In addition to structural damage, *C. trachomatis* was also demonstrated to modulate the immune response in human Sertoli cells, characterized by the activation of TLR3 alongside the down-modulation of downstream signaling pathways, namely NFκB and interferon regulatory factor (IRF)3 (Di Pietro et al., 2020a). Consequently, IFNs type-I and type-II, IL-1α and IL-6 were not produced, suggesting that *C. trachomatis* could evade the host immune-mediated killing, surviving in the cells and damaging the testicular tissue.

### Human Synovial Cells

The interaction between *C. trachomatis* and human synovial cells was investigated in 1998 by Rödel et al., showing, for the first time, the ability of *C. trachomatis* to infect fibroblast-like cells derived from biopsies of the synovial membrane (Rödel et al., 1998a). Then, *C. trachomatis* infection of synovial cells was also demonstrated to elicit the production of several pro-inflammatory cytokines, such as IL-6 and IFN-β (Rödel et al., 1998b).

Since then, few studies have further researched this interaction, detailing some cellular mechanisms underlying the synovial cell immune response to chlamydial infection. In particular, increased production of IRF1 and interferon-stimulated gene factor (ISGF)-3γ was observed in synovial cells infected with *C. trachomatis*, leading to the production of IFNβ as well as to the upregulation of Human Leukocyte Antigen (HLA)-1 gene expression (Rödel et al., 1999).

In recent years, the unique morphology, and the peculiar growth cycle of chlamydial inclusions in a primary human synovial cell have been described, and differed significantly from those in cervical epithelial cells. In particular, *C. trachomatis* was characterized by heterogeneous shape and size of inclusions and by a delayed developmental cycle, with late appearing infectious EBs (after 36 h.p.i.), as well as by a lower infection efficiency (Filardo et al., 2021).

The investigation of synovial cell immune response toward *C. trachomatis* evidenced a distinct profile characterized by the activation of TLR3 and TLR2 as well as of downstream signaling molecules, like ISG56 and Guanylate Binding Protein (GBP)1, interferon-inducible proteins involved in the cell-autonomous immunity against intracellular bacteria (MacMicking, 2012; Di Pietro et al., 2020b; Honkala et al., 2020). Nevertheless, the synovial cell response to *C. trachomatis* seemed ineffective in controlling the infection, suggesting its potential evasion of synovial cell inflammatory pathways (Filardo et al., 2021). Indeed, the increased expression of caspase-1 gene, in *Chlamydia* infected synovial cells, did not induce a parallel increase in the production of IL-6 as well as IL-1β and IL-18. A further interesting evidence is the observation that caspase activation played an important role in the intracellular growth of *C. trachomatis* in synovial cells, as demonstrated by decreased chlamydial replication after caspase inhibition (Filardo et al., 2021).

Differently, synovial cells were demonstrated to be able to control chlamydial infection when exposed to IFNγ, a well-known pro-inflammatory cytokine involved in the clearance of *C. trachomatis* genital infection. In particular, IFNγ was demonstrated to inhibit chlamydial growth decreasing caspase-1 gene expression while, at the same time, inducing TLR2 and ISG56 gene expression and IL1β, IL-18 and IL-6 production, suggesting the key role of IFNγ for the modulation of inflammatory and immune responses of synovial cells toward *C. trachomatis* (Di Pietro et al., 2020b).

## Discussion

**Figure 1:**
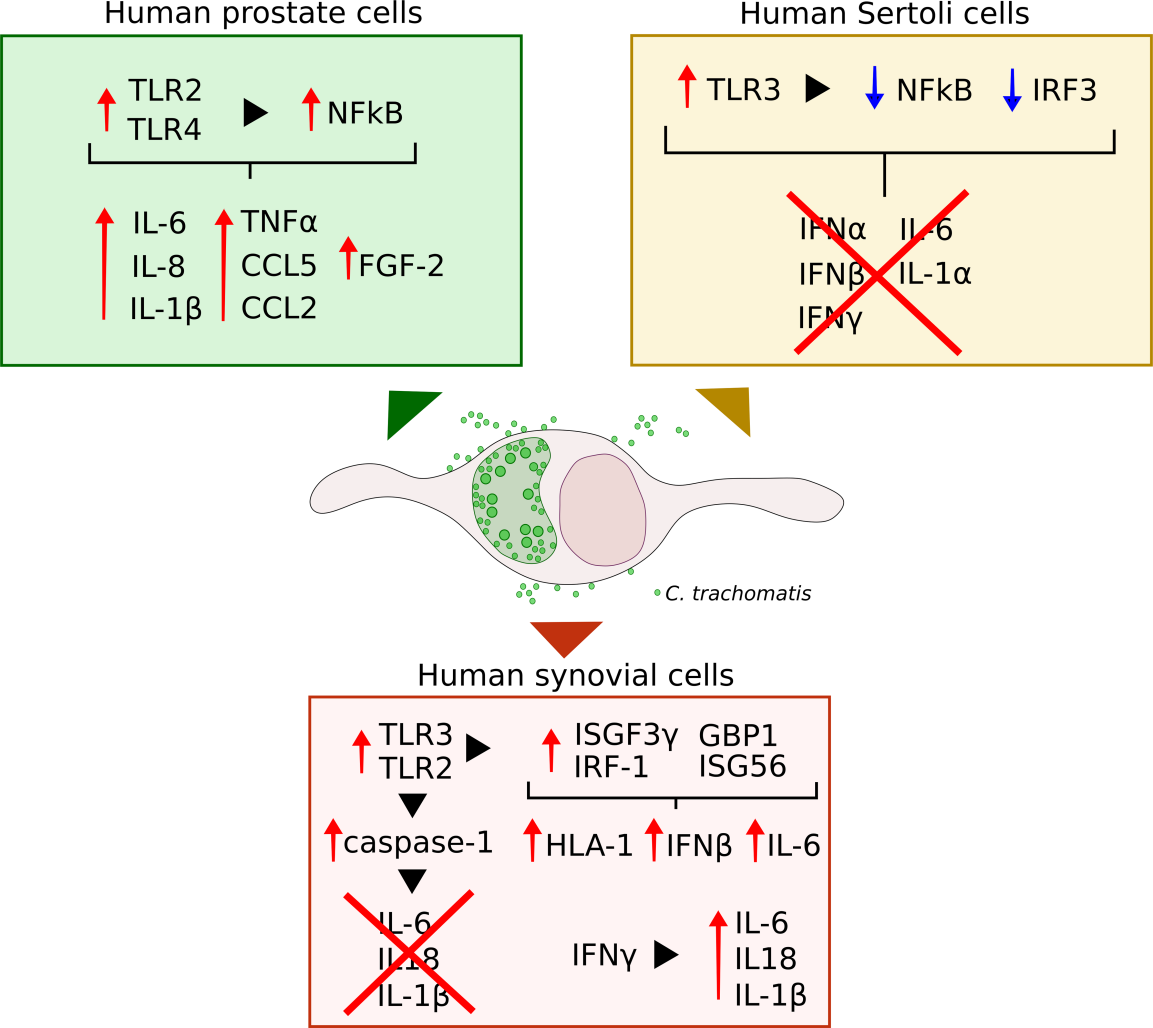
Schematic representation of the immune and inflammatory pathways elicited by *C. trachomatis* infection of human prostate epithelial, Sertoli and synovial cells.

Research to elucidate how *C. trachomatis* interact with specific cells of the prostate, male genital tract and joints is fundamental to understand how these interactions influence disease outcomes. To date, the available data provide insights regarding *C. trachomatis* growth within human prostate epithelial, Sertoli and synovial cells, and the related host-cell response pathways (**Figure 1**).

Human prostate epithelial cells showed a similar progression of chlamydial intracellular developmental cycle as that observed in cervical epithelial cells, alongside a comparable infection efficiency (Petyaev et al., 2019). By contrast, in Sertoli and synovial cells, the duration of the different *C. trachomatis* developmental stages, as well as the number of infectious EBs released from host cells, greatly differed as compared to those observed in cervical epithelial cells, routinely used for chlamydial research (Filardo et al., 2019b). The late appearances of infectious EBs toward the end of the developmental cycle suggests the presence of hostile cellular environment that may partially hinder chlamydial intracellular growth in these cells. In addition, the lower infection efficiency of *C. trachomatis*, previously described, in human Sertoli and synovial cells as compared to prostate epithelial cells, highlights that the prostate is particularly susceptible to *C. trachomatis* infection and, hence, could represent a trojan horse for the subsequent dissemination in the host, including the epididymis/testis or the joint.

Important differences were also observed in the immune and inflammatory host cell responses to *C. trachomatis* infection, with the activation of different molecular sensors, downstream signalling pathways and inflammatory signatures. Indeed, human prostate cells recognition of *C. trachomatis* by TLR2/TLR4 induced a pro-inflammatory state, highlighted by increased levels of IL-1β, IL-6, IL-8 and TNFα (Sellami et al., 2014; Petyaev et al., 2019). By contrast, TLR3-mediated sensing of chlamydial infection in human Sertoli cells did not elicit the activation of the related pathways, namely NFkB and IRF3, as well as the subsequent cytokine production. These results hint that in human prostate epithelial cells inflammation may play a key role in the pathogenesis of *C. trachomatis*-mediated tissue damage and prostate cancer progression. Instead, in Sertoli cells, *C. trachomatis* might induce direct cell-damage, as evidenced by the alteration of host-cell cytoskeleton, and, at the same time, remain within the cell for a long time, leading to a chronic infection. Indeed, in Sertoli cells, *C. trachomatis* appears to modulate the innate immune response, evading, hence, the host immune-mediate killing (Di Pietro et al., 2020a).

A further evasion strategy from host-cell defence pathways was also described in human synovial cells, as shown by *C. trachomatis* hijack of caspase-1 mediated inflammasome network, inhibiting the production of IL-6 as well as Il-1β and IL-18, involved in pyroptosis, a cellular defence mechanism against infectious agents (Man et al., 2017; Filardo et al., 2021). Surprisingly, earlier reports demonstrated the induction of a pro-inflammatory state in human synovial cells infected by *C. trachomatis*, evidenced by increased IL-6 levels (Rödel et al., 1998b; Rödel et al., 1999), whereas our recent studies evidenced a significant increase in pro-inflammatory cytokine levels following the exposure to IFNγ (Di Pietro et al., 2020b). These discordant results may be dependent on several factors, like, for example, the *C. trachomatis* serovar, since early reports used *C. trachomatis* serovar E, whereas the serovar D was investigated in recent studies (Rödel et al., 1998b; Filardo et al., 2021). The serotype D and E have also been used in studies involving Sertoli and prostate epithelial cells (Greenberg et al., 1985; Al-Mously and Eley, 2007; Filardo et al., 2019b; Di Pietro et al., 2020a); the lymphogranuloma venereum serovar L2 was used in prostate malignant cells (Sellami et al., 2014; Petyaev et al., 2019).

Overall, the path ahead is still long, and, in the future, more complex approaches with 3D cell cultures and organoids will be helpful for shedding light on the etiopathogenesis of *C. trachomatis*-mediated prostatitis, male infertility, and reactive arthritis, since many cell signalling pathways, that might have been elicited in response to chlamydial infection, are still unexplored.

## Author Contributions

MDP and RS conceived the manuscript. SF and FD performed literature review. SF, MDP, and RS wrote the original manuscript. SF, MDP, and RS revised the manuscript. SF and FD designed the Figure. All authors approved the submitted version.

## Funding

This research was funded by the University of Rome “Sapienza” to Prof. Rosa Sessa (grant RP11916B6AEB0D37).

## Conflict of Interest

The authors declare that the research was conducted in the absence of any commercial or financial relationships that could be construed as a potential conflict of interest.

## Publisher’s Note

All claims expressed in this article are solely those of the authors and do not necessarily represent those of their affiliated organizations, or those of the publisher, the editors and the reviewers. Any product that may be evaluated in this article, or claim that may be made by its manufacturer, is not guaranteed or endorsed by the publisher.
